# Transmissibility of tuberculosis among students and non-students: an occupational-specific mathematical modelling

**DOI:** 10.1186/s40249-022-01046-z

**Published:** 2022-12-02

**Authors:** Qiuping Chen, Shanshan Yu, Jia Rui, Yichao Guo, Shiting Yang, Guzainuer Abudurusuli, Zimei Yang, Chan Liu, Li Luo, Mingzhai Wang, Zhao Lei, Qinglong Zhao, Laurent Gavotte, Yan Niu, Roger Frutos, Tianmu Chen

**Affiliations:** 1 State Key Laboratory of Molecular Vaccinology and Molecular Diagnostics, School of Public Health, Xiamen University, Xiamen, Fujian People’s Republic of China; 2 CIRAD, URM 17, Intertryp, Montpellier, France; 3 Université de Montpellier, Montpellier, France; 4 Xiamen Center for Disease Control and Prevention, Xiamen, Fujian People’s Republic of China; 5 Jilin Provincial Center for Disease Control and Prevention, Changchun, Jilin People’s Republic of China; 6 Espace-Dev, Université de Montpellier, Montpellier, France; 7 Chinese Center for Disease Control and Prevention, 155 Changbai Road, Changping District, Beijing, China

**Keywords:** Tuberculosis, Transmission, Compartmental model, Occupational-specific dynamics, Student, Non-student, China

## Abstract

**Background:**

Recently, despite the steady decline in the tuberculosis (TB) epidemic globally, school TB outbreaks have been frequently reported in China. This study aimed to quantify the transmissibility of *Mycobacterium tuberculosis* (MTB) among students and non-students using a mathematical model to determine characteristics of TB transmission.

**Methods:**

We constructed a dataset of reported TB cases from four regions (Jilin Province, Xiamen City, Chuxiong Prefecture, and Wuhan City) in China from 2005 to 2019. We classified the population and the reported cases under student and non-student groups, and developed two mathematical models [nonseasonal model (Model A) and seasonal model (Model B)] based on the natural history and transmission features of TB. The effective reproduction number (*R*_*eff*_) of TB between groups were calculated using the collected data.

**Results:**

During the study period, data on 456,423 TB cases were collected from four regions: students accounted for 6.1% of cases. The goodness-of-fit analysis showed that Model A had a better fitting effect (*P* < 0.001). The average *R*_*eff*_ of TB estimated from Model A was 1.68 [interquartile range (IQR): 1.20–1.96] in Chuxiong Prefecture, 1.67 (IQR: 1.40–1.93) in Xiamen City, 1.75 (IQR: 1.37–2.02) in Jilin Province, and 1.79 (IQR: 1.56–2.02) in Wuhan City. The average *R*_*eff*_ of TB in the non-student population was 23.30 times (1.65/0.07) higher than that in the student population.

**Conclusions:**

The transmissibility of MTB remains high in the non-student population of the areas studied, which is still dominant in the spread of TB. TB transmissibility from the non-student-to-student-population had a strong influence on students. Specific interventions, such as TB screening, should be applied rigorously to control and to prevent TB transmission among students.

**Graphical Abstract:**

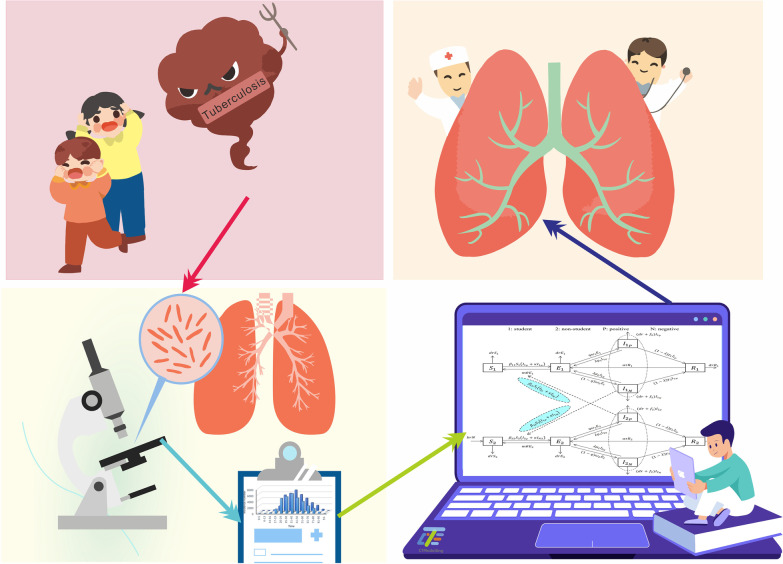

**Supplementary Information:**

The online version contains supplementary material available at 10.1186/s40249-022-01046-z.

## Background

Despite widespread implementation of control measures, including pharmaceutical therapy and vaccination, tuberculosis (TB) remains a major cause of disease and death in most high-burden countries. In 2021, most TB cases occurred in the 30 high-burden countries (87%), in which 8 countries account for two-thirds, with China (7.4%) ranking after India (28%) and Indonesia (9.2%) [1]. China is also on the three lists of high-burden countries for TB, HIV-associated TB, and multidrug resistant tuberculosis (MDR-TB) of the World Health Organization (WHO). Despite the difficulties that remain, such as the emergence of drug-resistant strains of *Mycobacterium tuberculosis* (MTB) and the coinfection of TB with the human immunodeficiency virus (HIV)/acquired immune deficiency syndrome (AIDS), the global incidence of tuberculosis is estimated to slowly decline by 1.6% per year, far from the 4–5% estimated to be required to reach the objectives of the WHO’s End TB Strategy [2]. Due to the emergence of the COVID-19 pandemic, there is a large global drop in people newly diagnosed with TB and reported in 2020, compared to 2019[3].

In China 2021, the number of reported TB cases is ranked second highest after viral hepatitis, and in terms of death is the second highest after AIDS [4]. There are about 250 million students in China (about 20% of the population). The reported TB cases in students account for about 4–6% of the total reported TB cases [5]. TB cases in the 15–24-year age group accounted for about 85% of the total reported TB cases in students, which means the number of TB cases in high school and college students is higher, especially in the 18-year-old age group [6–8]. When MTB spreads in schools, it can be transmitted rapidly and have a major impact on young people simply because of cluster. Therefore, it is one of the reasons why school TB outbreaks have been reported frequently in China, despite the steady global decline of the TB incidence trend [9–13]. Moreover, MDR-TB outbreaks have also been reported in schools, making TB control in schools much more difficult [14, 15].

Theoretical epidemiology, also known as the mathematical model of epidemiology, uses mathematical formulas to express the law of disease prevalence explicitly and quantitatively between cause, host and environment, and to theoretically explore the effects of different control measures. Mathematical modelling has become a powerful tool for analysing epidemiological characteristics [16], which is used to reveal the characteristics of the internal spread of infectious diseases. Transmission dynamic models are commonly used in infectious disease models, including Susceptible-infectious-recovered model, Susceptible-exposed-symptomatic-recovered (SEIR) model, and Autoregressive integrated moving average model. Some studies use models to analyse TB, such as TB intervention assessments [17], analysis of vaccine control effectiveness [18, 19], and TB treatment [20–23]. Different models have been developed to treat latent TB infections (LTBI) that incorporate certain factors such as drug-resistant strains [24], coinfection with HIV [25], and TB reinfection [26], and to study the epidemiology of TB [27]. Specific targeted sub-populations have been defined, including age-specific subgroups [28], adults and children [29], and smokers and non-smokers [30]. However, only a few studies have used occupational mathematical models to study TB transmission in China. The construction of TB models which are used to explore the dynamics of TB transmission between students and non-students is unclear.

The prevention and control of TB in schools has been improved with the efforts of medical personnel staff at all levels. In the past 10 years, control measures have been continuously strengthened and improved, but the transmission characteristics of TB in schools are still unclear. The aim of this study is to establish a mathematical model of TB between students and non-students, to analyse and explore the transmissibility of MTB in schools, and then to take reasonable and effective measures to control TB in schools.

## Methods

### Study design

In this study, based on the reported and observed morbidity characteristics, we developed a SEIR model with two occupational groups (students and non-students). We investigated the role of occupation in the transmission process and evaluated feasible control strategies to achieve the objectives outlined in the WHO End TB Strategy [3]. Furthermore, this study classified active TB patients into high or low transmissibility groups according to their pathogenic status [31].

Firstly, in this study, a dataset was constructed, including basic information (sex, age, occupation, and location) and case classification (positive and negative cases of pathogen). Demographic data was obtained from the Chinese Statistical Yearbook [32–35], including the total population, the total student population, birth rate, and the death rate for each city (Additional file 1: Table S1).

Secondly, two mathematical models (Models A and B refer to non-seasonal and seasonal models, respectively) were constructed to simulate the reported TB cases of the four regions in China, based on the natural history and seasonality of TB. In each model, we divided the collected data into four subpopulations of active diseases in two dimensions. The first dimension for all calculations and outputs was the occupation of students (_1_ subscript) or non-students (_2_ subscript), while the second dimension was the pathogenic status, including pathogen positive (_p_ subscript) and pathogen negative (_n_ subscript) pulmonary disease. In addition, goodness-of-fit was conducted to evaluate the effectiveness of model fitting.

**Fig. 1:**
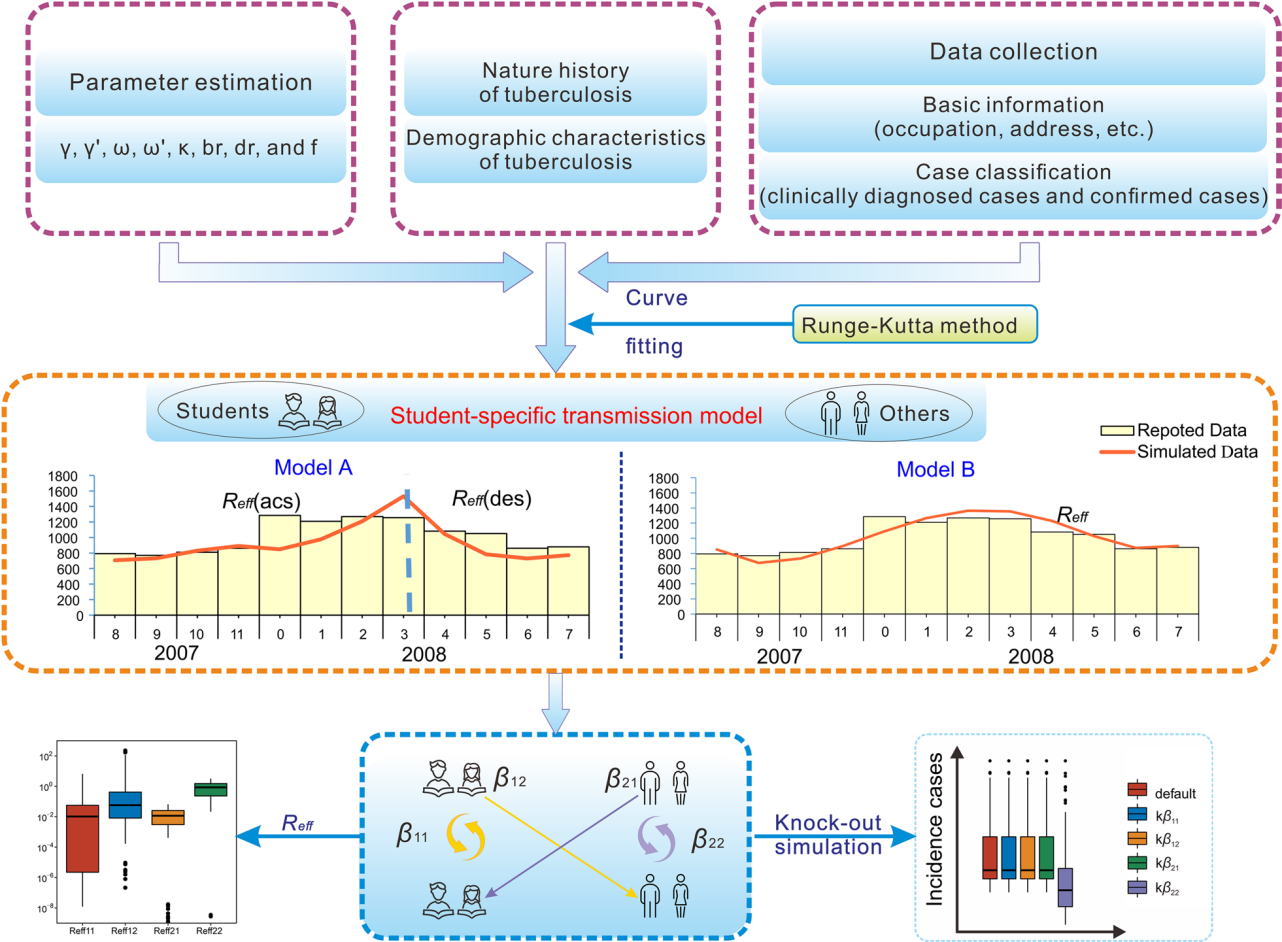
Study design for analysing the transmissibility of TB among students and non-students. The four subscripts are denoted as follows: TB transmission in student groups (_11_ subscript), TB transmission of student-to-non-student groups (_12_ subscript), TB transmission in non-student groups (_22_ subscript), TB transmission of non-student-to-student groups (_21_ subscript)

Finally, we simulated the sub-population-to-sub-population transmission process, to determine the combination with the most distinctive impact, via calculating effective reproduction number (*R*_*eff*_) and performing knock-out analysis. This enabled the formulation of effective and targeted control measures for TB transmission in China, in accordance with occupation-specific prevention and control (Fig. 1).

### Data collection

**Fig. 2:**
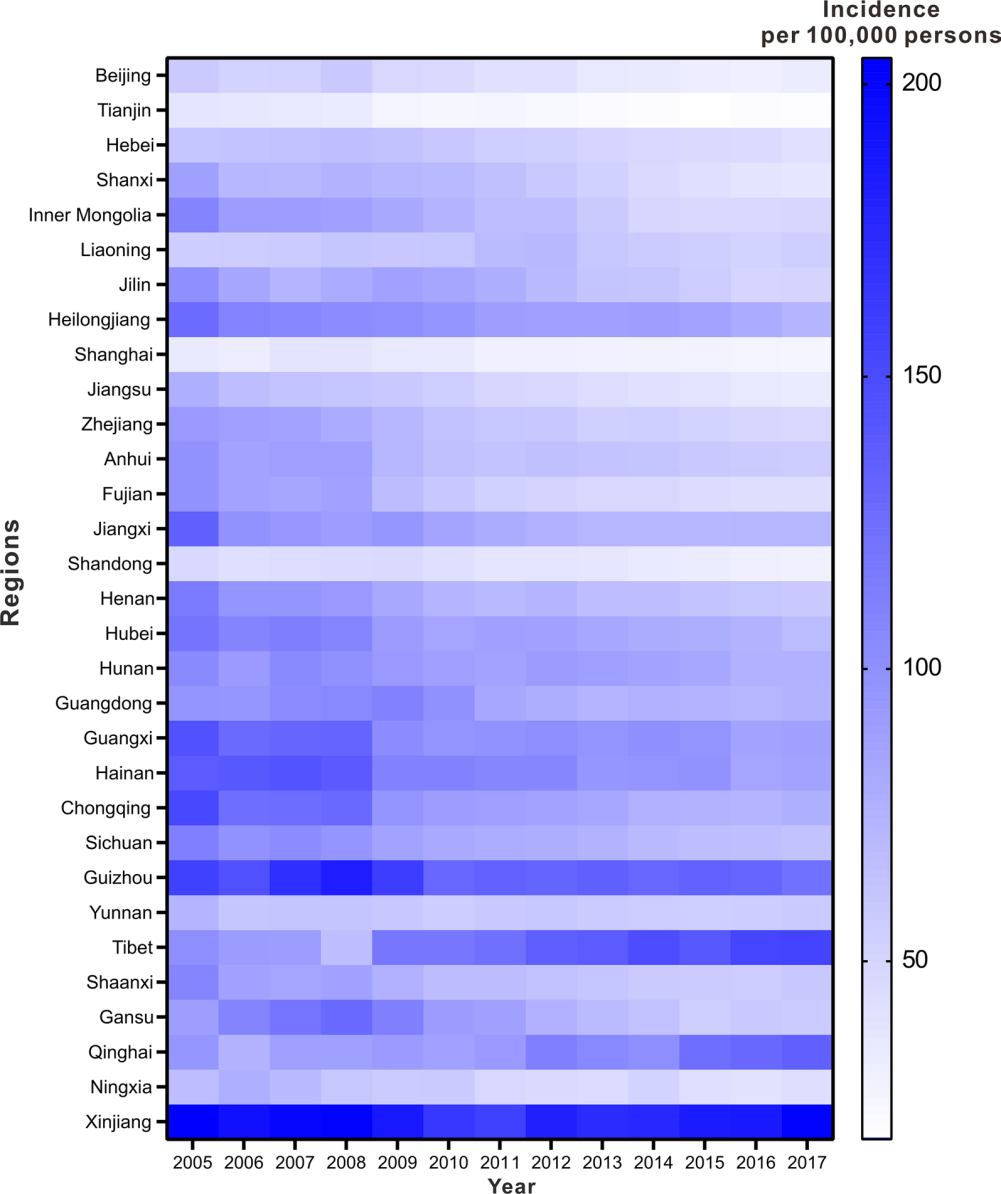
Regional and national distribution of reported TB incidence. Reported TB incidence in different regions (not included Hong Kong, Macao, and Taiwan) in China from 2005 to 2017

We collected year-based TB incidence data from the China Public Health Science Data Center (http://www.phsciencedata.cn/Share/index.jsp) from January 1, 2005 to December 31, 2017 for each province in China (not included Hong Kong, Macao, and Taiwan) [36]. After we calculated the average annual incidence rate and plotted the incidence map (Fig. 2), we found an inequality in the disease burden.

Considering the geographic position, the TB incidences in four regions (located in the north, south, southwest, and middle of China) are average incidences compared to those of the other regions in China, which is consistent with the population distribution [37], which means that the selection can better reflect the TB epidemiological characteristics in geographical differences.

This study collected data on reported TB cases, populations, and areas in four regions [Jilin Province, Wuhan City in Hubei Province, Chuxiong Yi Autonomous Prefecture (Chuxiong Prefecture) in Yunnan Province, and Xiamen City in Fujian Province] (Additional file 1: Table S1), which are from the Health Commission of each region, the Statistics Bureau of each region and data mentioned in some researches [38, 39], etc. Therefore, the TB results from these four regional analyses are effectively representative of TB epidemiological characteristics in China.

### Classification of TB patients

Reported TB cases included in this study consisted of laboratory confirmed pulmonary tuberculosis (PTB), and clinical diagnostic PTB. Since the Chinese government implemented the National Notifiable Disease Surveillance System (NNDSS) for infectious diseases in 2004, the diagnostic criteria for TB has changed several times (“WS288–2008 Diagnosis of tuberculosis” [40] with the adjusted notice in 2017 [41], and the “WS 288–2017 Diagnosis of Tuberculosis” [42] and the “WS 196–2017 Classification of Tuberculosis” standards [43], with the adjusted notice in 2018 [31]). All TB cases were classified on the base of the following criteria. The confirmed PTB cases were denoted as people with possible PTB symptoms, such as a continuous cough for more than 2 weeks, hemoptysis, and night sweat, and confirmed by a sputum smear and/or a sputum culture with the result of detectable acid-fast bacilli or positive result from a rapid molecular diagnostic instrument (e.g., GeneXpert). The clinical diagnosis of PTB was defined as people with obviously abnormal chest radiography along with no curative effect from anti-inflammatory treatment under the circumstance of negative results from laboratory tests or absence of related results [44–46].

The PTB cases are classified as follows, based on pathogenic findings: sputum smear positive, sputum smear negative, sputum culture positive, sputum culture negative, molecular biology positive, and without sputum PTB [42]. In the latest notice published in 2018, the classification of TB cases, which must be reported in the NNDSS, was adjusted to “pathogen positive (including sputum smear positive and only sputum culture positive PTB)”, “pathogen negative (including sputum smear negative PTB)”, “rifampicin resistant”, “no pathogenic findings (including without sputum PTB and tuberculous pleurisy” [31]. We have reclassified all historical data according to the new classification notice for consistency (see detail in Additional file 2: Table S2).

### Diagnosis criteria of PTB patients

The diagnosis of PTB is based on a pathogenic examination (including bacteriology and molecular biology), combined with epidemiological history, clinical manifestations, chest imaging, relevant auxiliary examinations, differential diagnosis, and other comprehensive analyses [47]. Pathogenic and pathological results were used as the basis for diagnosis. Therefore, the following inclusion criteria were TB cases with pathogen positive [“positive cases with MTB detected by sputum smear, culture-confirmed or molecular biology (nucleic acid of MTB)”] and negative [“TB cases without MTB detected (including patients with negative sputum smear and without sputum)”]. The rifampicin resistance category was officially reported in 2019 and represented a small percentage (< 5%) of the total data collected. Therefore, to maintain the consistency of the overall data, we excluded these data from the analysis.

### Occupational-specific transmission model of TB

Based on the model, the total population (*N*) was divided into the following five compartments: susceptible population (*S*), exposed population (or low-risk latent tuberculosis infection, LTBI) (*E*), pathogen positive tuberculosis population (*I*_*p*_), pathogen negative tuberculosis population (*I*_*n*_), and recovered or removed population (*R*).

1. Susceptible population (*S*): people who have not been exposed to MTB or those who experienced self-clearance by their own immune system. The latter is a state in which the bacteria in the body cannot replicate to the extent that self-clearance occurs due to the strong immunity of the body after exposure, a state in which the body has a sustained immune response to MTB antigen stimulation.
2. Exposed population (or low-risk LTBI) (*E*): A susceptible population is exposed to MTB through contact with a highly infectious or less infectious population and is in an MTB carrier state but is temporarily noninfectious.
3. Pathogen positive TB population (*I*_*p*_): positive cases with MTB detected by sputum smear, culture-confirmed, or molecular biology (nucleic acid of MTB).
4. Pathogen negative TB population (*I*_*n*_): TB cases without MTB detected (including patients with negative sputum smear and no sputum), with low infectiousness.
5. Recovered or removed population (*R*): This is a state of cure or recovery, noninfectious and asymptomatic, referring to the population undergoing successful treatment, including the treatment success for the “pathogen negative” population and the “pathogen positive” population (both the cured and the treatment success population).

Based on the natural history of TB, we developed a mathematical model Susceptible-exposed-symptomatic (pathogen positive)-symptomatic (pathogen negative)-recovered (*SEI*_*p*_*I*_*n*_*R*) model to investigate the transmission process of TB. The proposed *SEI*_*p*_*I*_*n*_*R* model is based on the following facts and assumptions:

1. Births and natural deaths change the total population (*N*); the birth rate and the death rate are *br* and *dr*, respectively. The entire birth population enters group 2(the non-student group).
2. This population is generally susceptible to MTB infection. When an infected individual is exposed, the exposed population (*E*) progresses to the active TB-infected population (*I*) at a rate of *β*. Since the transmissibility of the pathogen positive TB population (*I*_*p*_) is higher than the pathogen negative TB population (*I*_*n*_), the transmissibility of *I*_*n*_ is set to be a *к* times (*к* < 1) compared to *I*_*p*_.
3. Approximately 5–10% of the exposed population (E) infected with MTB will develop symptoms and become *I*_*p*_ or *I*_*n*_; both belong to the active TB-infected population and will receive treatment. Most exposed people do not develop symptoms, but undergo a process of self-clearance and become a susceptible population (*S*). If *E* progresses to *I*_*p*_ at a rate of *ω*_*1*_ (incubation period coefficient) with a scale factor of *q*, and *E* progresses to *I*_*n*_ at a rate of *ω*_2_ (latent period coefficient) with a scale factor of (1-*q*). The progression from *E* to *S* occurs at a rate of *θ* (early self-clearance rate) on a scale factor of *m*. At time *t*, the progression from *E* to *I*_*p*_, from *E* to *I*_*n*_ and from *E* to *S* is proportional to the number of exposed populations, which is *qω*_1_*E*, (1-*q*)*ω*_2_*E*, and *mθE*, respectively.
4. Studies have shown that the proportion of patients with TB cured by the directly observed treatment and short course chemotherapy (DOTS) who require retreatment in the next 1–2 years is 2 to 7% [48, 49]. Patients who are retreated can be broadly divided into two categories: those who were not successfully cured following treatment, and those who relapsed after being cured.
5. There were two outcomes for the *I*_*p*_ compartment: First, a certain proportion  of treatment success individuals (1-*λ*) transform into a recovered or removed population (*R*), while another proportion of treatment failure individuals (*λ*) transform into an exposed population (*E*). At time *t*, the rate of development from *I*_*p*_ to *R*, which is proportional to the *I*_*p*_ population, is given as (1-*λ*)*γ*_1_*I*_*p*_, while the rate of development from *I*_*p*_ to *E*, which is proportional to the *E* population, is given as *λμ*_1_*I*_*p*_. *γ*_1_ is the removal coefficient, whereas *μ*_1_ is the coefficient of development from *I*_*p*_ to *E*. Similarly, the rate of development to *R* in the *I*_*n*_ compartment is given as (1-*λ*)*γ*_2_*I*_*n*_, while the rate of development to *E* is given as *λμ*_2_*I*_*n*_. *γ*_2_ is the removal coefficient and *μ*_2_ is the coefficient of development of *I*_*n*_ to *E*.
6. The people in the *I*_*p*_ and *I*_*n*_ compartments recover or are removed (*R*) after successful treatment (completion of treatment for *I*_*n*_ and *I*_*p*_ [50]). Reinfection occurs after the completion of treatment or cured, that is, the active TB-infected population (*I*_*p*_, *I*_*n*_) returns to the exposed population (*E*).
7. Reactivation (or relapse) is often associated with immunodeficiency, such as the onset of disease due to HIV/TB coinfection   or low resistance, such as severe cold. If people in the *R* compartment develop into *E* with a relapse ratio *a* where *τ* represents the relapse coefficient, the rate of development from *R* to *E* at time *t* is proportional to *R*, which is *aτR*.
8. The pathogen positive TB population (*I*_*p*_) and the pathogen negative TB population (*I*_*n*_) die of disease, in addition to natural deaths. Suppose the fatality rates for *I*_*p*_ were *f*_1_ and that for *I*_*n*_ were *f*_2_; then, at time t, the death rates for *I*_*p*_ and *I*_*n*_ are *f*_1_*I*_*p*_ and *f*_2_*I*_*n*_, respectively.
9. The student population was set as *S*_1_, *E*_1_, *I*_*p*1_, *I*_*n*1_, and *R*_1_, whereas the non-student population was set as *S*_2_, *E*_2_, *I*_*p*2_, *I*_*n*2_, and *R*_2_. Interactions were observed between students and non-students. We defined the relative transmission rate of student-to-student as *β*_11_, non-student-to-non-student as *β*_22_, student-to-non-student as *β*_12_, and non-student-to-student as *β*_21_. Therefore, the number of newly emerging infections was *β*_11_*S*_1_(*I*_*p*1_ + к*I*_*n*1_) from the student-to-student population, *β*_22_*S*_2_(*I*_*p*2_ + к*I*_*n*2_) from the non-student-to-non-student population, *β*_12_*S*_2_(*I*_*p*1_ + к*I*_*n*1_) from the student-to-non-student population, and *β*_21_*S*_1_(*I*_*p*2_ + к*I*_*n*2_) from the non-student-to-student population.

**Fig. 3:**
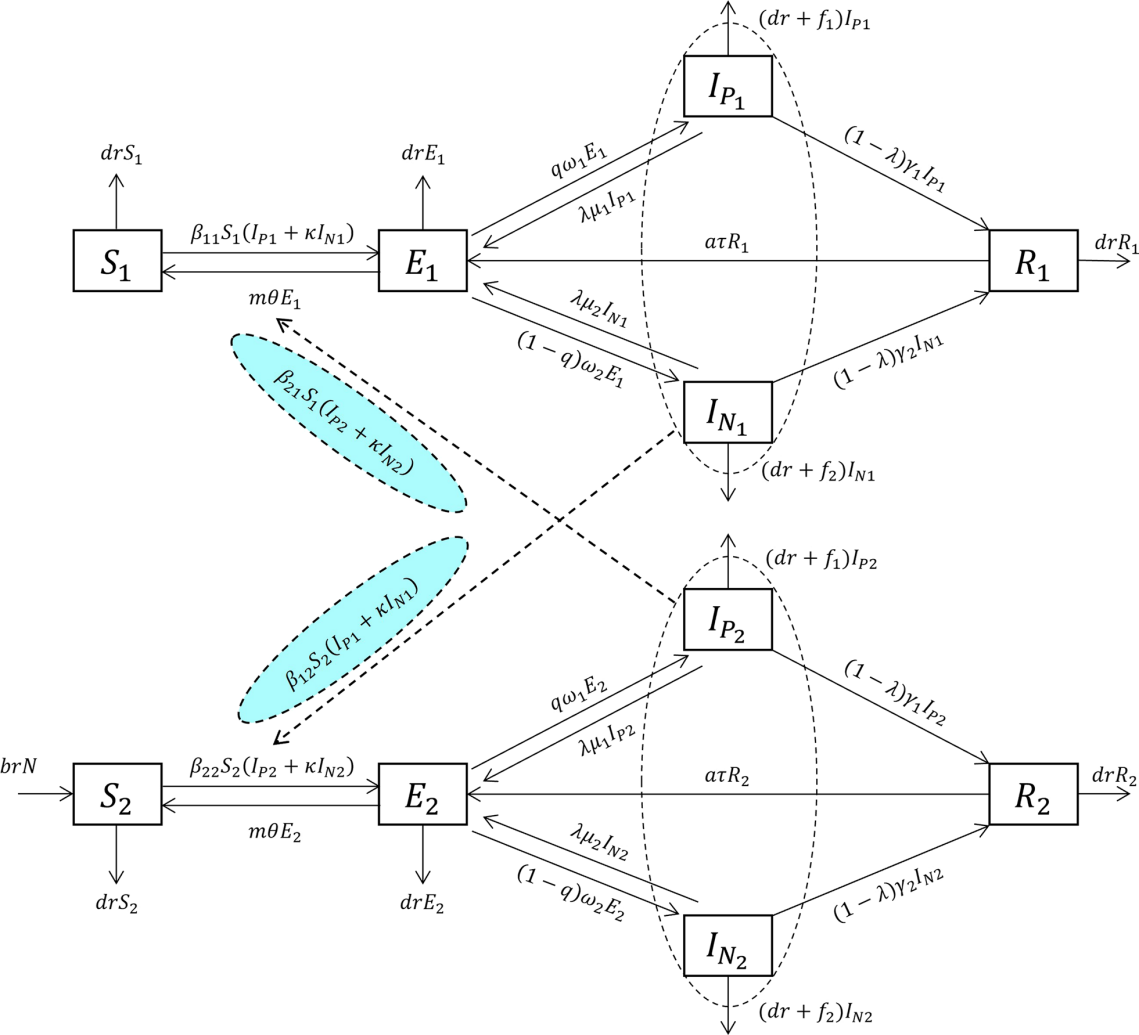
Flowchart of the *SEI*_*p*_*I*_*n*_*R* model. The four occupational compartments are denoted as follows: pathogen positive students (I_p1_ subscript), pathogen positive non-students (I_p2_ subscript), pathogen negative students (I_n1_ subscript) and pathogen negative non-students (I_n2_ subscript)

A framework diagram of the *SEI*_*p*_*I*_*n*_*R* model is shown in Fig. 3. The mathematical expression of the differential equation of the *SEI*_*p*_*I*_*n*_*R* model are as follows:

$$\frac{\mathrm{d}{S}_{1}}{\mathrm{d}t}=-{\beta }_{11}{S}_{1}\left({I}_{p1}+\kappa {I}_{n1}\right)+m\theta {E}_{1}-{\beta }_{21}{S}_{1}\left({I}_{p2}+\kappa {I}_{n2}\right)-dr{S}_{1}$$

$$\frac{\mathrm{d}{E}_{1}}{\mathrm{d}t}={\beta }_{11}{S}_{1}\left({I}_{p1}+\kappa {I}_{n1}\right)-m\theta {E}_{1}+{\beta }_{21}{S}_{1}\left({I}_{p2}+\kappa {I}_{n2}\right)-dr{E}_{1}-q{\omega }_{1}{E}_{1}+\lambda {\mu }_{1}{I}_{p1}-\left(1-q\right){\omega }_{2}{E}_{1}+\lambda {\mu }_{2}{I}_{n1}+\alpha \tau {R}_{1}$$

$$\frac{\mathrm{d}{I}_{p1}}{\mathrm{d}t}=q{\omega }_{1}{E}_{1}-\lambda {\mu }_{1}{I}_{p1}-\left(1-\lambda \right){\gamma }_{1}{I}_{p1}-{(dr+f}_{1}){I}_{p1}$$

$$\frac{\mathrm{d}{I}_{n1}}{\mathrm{d}t}=\left(1-q\right){\omega }_{2}{E}_{1}-\lambda {\mu }_{2}{I}_{n1}-\left(1-\lambda \right){\gamma }_{2}{I}_{n1}-(dr+{f}_{2}){I}_{n1}$$

$$\frac{\mathrm{d}{R}_{1}}{\mathrm{d}t}=\left(1-\lambda \right){\gamma }_{1}{I}_{p1}+\left(1-\lambda \right){\gamma }_{2}{I}_{n1}-\alpha \tau {R}_{1}-dr{R}_{1}$$

$$\frac{\mathrm{d}{S}_{2}}{\mathrm{d}t}=brN-dr{S}_{2}-{\beta }_{22}{S}_{2}\left({I}_{p2}+\kappa {I}_{n2}\right)+m\theta {E}_{2}-{\beta }_{12}{S}_{2}({I}_{p1}+\kappa {I}_{n1})$$

$$\frac{\mathrm{d}{E}_{2}}{\mathrm{d}t}={\beta }_{22}{S}_{2}\left({I}_{p2}+\kappa {I}_{n2}\right)-m\theta {E}_{2}+{\beta }_{12}{S}_{2}({I}_{p1}+\kappa {I}_{n1})-dr{E}_{2}-q{\omega }_{1}{E}_{2}+\lambda {\mu }_{1}{I}_{p2}-\left(1-q\right){\omega }_{2}{E}_{2}+\lambda {\mu }_{2}{I}_{n2}+\alpha \tau {R}_{2}$$

$$\frac{\mathrm{d}{I}_{p2}}{\mathrm{d}t}=q{\omega }_{1}{E}_{2}-\lambda {\mu }_{1}{I}_{p2}-\left(1-\lambda \right){\gamma }_{1}{I}_{p2}-{f}_{1}{I}_{p2}-dr{I}_{p2}$$

$$\frac{\mathrm{d}{I}_{n2}}{\mathrm{d}t}=\left(1-q\right){\omega }_{2}{E}_{2}-\lambda {\mu }_{2}{I}_{n2}-\left(1-\lambda \right){\gamma }_{2}{I}_{n2}-{f}_{2}{I}_{n2}-dr{I}_{n2}$$

$$\frac{\mathrm{d}{R}_{2}}{\mathrm{d}t}=\left(1-\lambda \right){\gamma }_{1}{I}_{p2}+\left(1-\lambda \right){\gamma }_{2}{I}_{n2}-\alpha \tau {R}_{2}-dr{R}_{2}$$

### Parameter estimation

Fourteen parameters were obtained from references or actual data in this model: birth rate (*br*)*,* death rate (*dr*)*,* transmission relative rate (*β*)*,* proportion of early clearance (*m*)*,* rate of early clearance (*θ*)*,* transmission relative rate between pathogen negative and positive TB population (*к*)*,* proportion of exposed to TB population (*q*)*,* rate of exposed to TB population (*ω*)*,* proportion of treatment failure (*λ*)*,* rate from TB population to exposed population (*μ*)*,* TB population removal coefficient (*γ*)*,* Case fatality rate of TB population (*f*)*,* recurrence ratio- proportion of recovered or removed population developing into exposed population (*a*)*,* and reciprocal of time to recurrence rate at which recovered or removed population progresses to exposed population (*τ*)*.*

Parameter *β* was derived from the curve-fitting results. Some parameters (*br, dr,* and *q*) were obtained from actual data, while other parameters were obtained from the literature. The description of each variable and parameter in this model is detailed in Table 1.

1. Early self-clearance (early clear) was defined as a persistent negative interferon-gamma release test (IGRA) (patients with pathogenically positive TB were tested at baseline and after 14 weeks). Studies in Indonesia have shown that early self-clearance is 25% [51]. The time to self-clearance was set at 14 weeks, which is the interval between the two IGRA tests; thus *θ* = 1/ (14/4) = 0.286.
2. Treatment failure: The WHO 2021 TB report [3] showed the treatment success rate was 95.9% in 2019 and 95.7% in 2020. Previous reports revealed that this value did not change much between 2000 and 2020. Therefore, we considered the treatment success rate as 95% and set the treatment failure rate (*λ*) to 5%, that is *λ* = 0.050. The conventional treatment course was 6 months. Therefore, the time to complete the treatment was set as 6 months, that is, *μ*1 = *μ*2 = 1/6 = 0.167 [52].
3. Relapse: Studies [53–55] in China showed the relapse rate was 5.3–6.9%. Therefore, the median was chosen and the relapse proportion was set at *a* = 0.062 (recurrence ratio). A domestic study [53] showed that the median time from the first attack to relapse in TB patients was 1.3 years [interquartile range (IQR) 0.6–2.8 years]. Therefore, the relapse rate was established at 1/(1.3*12), i.e., *τ* = 0.064 (reciprocal of time to recurrence).
4. The transmissibility coefficient of *I*_*n*_ relative to *I*_*p*_ was set as κ to 0.2 [56] with reference to the actual data and the relevant literature.
5. After approximately two weeks of effective treatment, TB cases with a nondrug-resistant active infection usually do not remain infectious to others and become low in infection status [57]. Short-course (3-to 4-month) rifamycin-based treatment regimens are preferred over longer-course (6 to 9 months) isoniazid monotherapy for the treatment of low-infection cases of TB [8]. Therefore, we set the duration of the illness at 14 weeks (average value 3–4 month), *γ*_1_ = *γ*_2_ = 1/(14/4) = 0.286.
6. The birth rate (*br*) and the death rate (*dr*) for each year in each region were obtained from the statistical offices of each study area.

**Table 1:** The description and features of estimated parameters

Parameter	Description	Unit	Value	Source
*br*	Birth rate	1	Null	Reported data
*dr*	Death rate	1	Null	Reported data
*β*	Transmission relative rate	Per person. per month	Null	Curve fitting
*β*_11_	Transmission relative rate among students	Per person. per month	Null	Curve fitting
*β*_22_	Transmission relative rate among non-students	Per person. per month	Null	Curve fitting
*β*_12_	Transmission relative rate from students to non-students	Per person. per month	Null	Curve fitting
*β*_21_	Transmission relative rate from non-students to students	Per person. per month	Null	Curve fitting
*κ*	Transmission relative rate between population *I*_*n*_ and population *I*_*p*_	1	0.2	Reference [56]
*m*	Proportion of early clearance	1	0.25	Reference [51]
*θ*	Rate of early clearance	Per month	0.286	Reference [51]
*q*	Proportion from *E* to *I*_*p*_	1	Null	Reported data
*1-q*	Proportion from *E* to *I*_*n*_	1	Null	Reported data
*ω*_1_	Rate from *E* to *I*_1_	Per month	0.667	Reference [93]
*ω*_2_	Rate from *E* to *I*_2_	Per month	0.667	Reference [93]
*λ*	Proportion of treatment failure	1	0.05	Reference [3]
*μ*_1_	Rate from *I*_1_ to *E* (reciprocal time to retreatment)	Per month	0.167	Reference [52]
*μ*_2_	Rate from *I*_2_ to *E* (reciprocal time to retreatment)	Per month	0.167	Reference [52]
*γ*_1_	*I*_1_ removal coefficient	Per month	0.286	Reference [57]
*γ*_2_	*I*_2_ removal coefficient	Per month	0.286	Reference [57]
*f*_1_	Case fatality rate of *I*_1_	1	0.1284	References [94, 95]
*f*_2_	Case fatality rate of *I*_2_	1	0.1284	Reference [94, 95]
*a*	Proportion of *R* developing into *E* (recurrence ratio)	1	0.062	References [53–55]
*τ*	Rate at which *R* progresses to *E* (reciprocal of time to recurrence)	Per month	0.064	Reference [53]

*E* for the exposed population (or low-risk latent tuberculosis infection, LTBI), *I*_*p*_ for pathogen positive tuberculosis population, *I*_*n*_ for pathogen negative tuberculosis population, *I*_1_ for student tuberculosis population, *I*_2_ for non-student tuberculosis population, and* R* for recovered or removed population

### Transmissibility index

The basic reproduction number (*R*_0_) is an important parameter for determining the infectiousness of a disease. *R*_0_ refers to the number of new cases expected from an infected case in a susceptible population during an average infectious period. We set *R*_*eff*_ as the evaluation index, which denotes *R*_0_ after intervention measures were taken, to evaluate the impact of intervention measures on the relative transmissibility of MTB in the population.

In this study, *R*_*eff*_ was calculated using the next-generation matrix method, and all source codes are accessible at GitHub (https://github.com/rorschachkwok/tb_reff). In this study, *R*_*eff*1_ represents the transmissibility of the population of students with active TB [sum of transmissibility from student cases to student cases (*R*_*eff*11_) and from student cases to non-student cases (*R*_*eff*12_)], while *R*_*eff2*_ represents the transmissibility of the population of non-student active TB cases [sum of transmissibility from non-student cases to non-student cases (*R*_*eff*22_) and from non-student cases to student cases (*R*_*eff*21_)].

### Simulation methods and statistical analysis

Berkeley Madonna 8.3.18 (developed by Robert Macey and George Oster of the University of California in Berkeley. Copyright ©1993–2001 Robert I. Macey & George F. Oster) was used to fit the curves of the incidence data. The estimated model coefficients and the simulation of the intervention effects were also generated using this software. The curving fit was performed using the fourth order Runge–Kutta method to obtain the key parameter values: student-to-student (*β*_11_), non-student-to-non-student (*β*_22_), student-to-non-student (*β*_12_), and non-student-to-student (*β*_21_) transmission rates.

To consider the potential seasonality transmission of TB, although seasonality remains unclear, we developed two models in this study, which are described as follows:

**Model A**: seasonality excluded.

In Model A, the epidemic curve for each year was divided into ascending and descending periods according to the characteristics of the reported number of TB cases (Fig. 1). The *SEI*_*p*_*I*_*n*_*R* model without seasonality was adopted to fit the data in each period, and the corresponding transmission relative rates (*β*, *β*_11_, *β*_12_, *β*_22_, and *β*_21_), the ascending and descending *R*_*eff*_ (*R*_*eff*(*asc*)_ and *R*_*eff*(*des*)_, respectively) were calculated.

**Model B**: seasonality included.

In Model B, we used a seasonality function in the *SEI*_*p*_*I*_*n*_*R* model to fit the reported TB epidemic curve (Fig. 1), which is shown as follows:

$${\beta }_{t}={\beta }_{0}\left(1+sin\left(\frac{2\pi \left(t-c\right)}{T}\right)\right)$$

In this equation, *β*_*t*_, *β*_0_, *c*, and *T* refer to the transmission rate at time t, the transmission rate at time = 0, the correcting value of time (month) and the potential seasonality cycle, respectively.

The goodness-of-fit test was performed between the fitted results and the collected data by calculating the *R*^2^ and *P* values. Key parameters (*β*_11_, *β*_12_, *β*_22_, *β*_21_) were knocked out, and the cumulative number of cases was calculated to assess the main parameter affecting transmissibility. SPSS Statistics for Windows, version 13.0 (SPSS Inc., Chicago, Ill, USA) was used to perform statistical analyses. The coefficient of determination (*R*^*2*^) was used to evaluate the curve fitness.

## Results

### Epidemiology of tuberculosis in the four regions

**Fig. 4:**
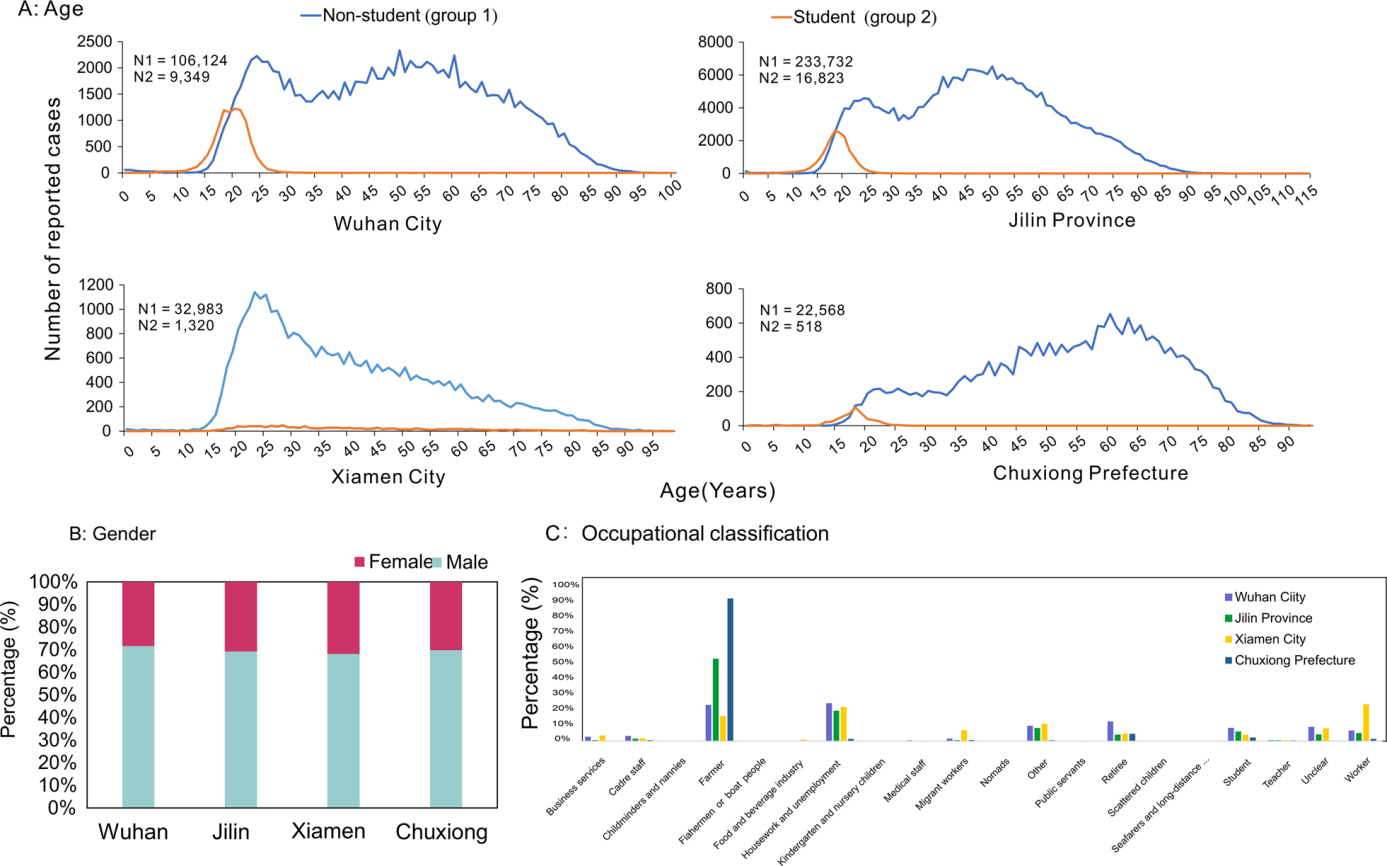
Age, gender, and occupation distributions: **A** Wuhan City, **B** Jilin Province, **C** Xiamen City, and **D** Chuxiong Prefecture

The age range of the TB patients was between 15 and 90 years, with two peaks in the incidence of TB: a large and a small peak in the age groups 35–90 and 15–35, respectively. In Wuhan City, Jilin Province and Chuxiong Prefecture, there were two age distribution peaks of non-student TB patients (15–35 and 45–60 years group), while in Xiamen City, there was only one peak (15–35 years group). Student patients with TB were among 15–25 year group (Fig. 4A). Most patients with TB in the four regions were male, with a male-to-female ratio of 7∶3 (Fig. 4B). The Chinese Infectious Disease Report Card categorises cases into 18 categories, and the top six occupations (88.4% of the total cases) in the four regions were: farmers, housework and unemployment, others, workers, students, and retirees. Among these four regions, the top three occupations of TB patients in Wuhan were domestic and unemployed (23.2%), farmers (22.2%), and retirees (12.1%). The top three occupations of TB patients were farmers (50.3%), domestic and unemployed (18.6%), and others (8.0%) in Jilin Province; workers (22.5%), farmers (15.3%), and others (10.6%) in Xiamen; farmers (87.2%), retirees (4.5%), and students (2.2%) in Chuxiong Prefecture (Fig. 4C). The ranking of students with TB was sixth, sixth, eighth and third in Wuhan, Jilin, Xiamen, and Chuxiong Prefecture, respectively.

**Fig. 5:**
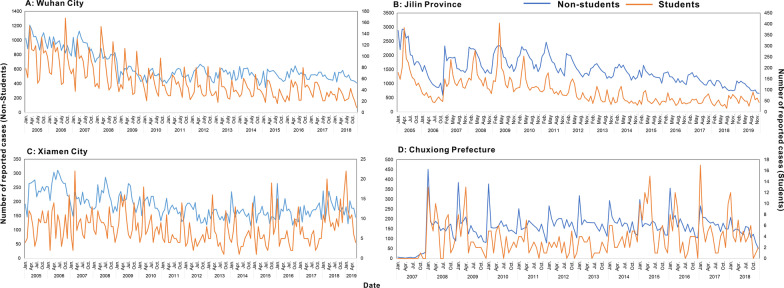
Temporal distribution by month: **A** Wuhan City, **B** Jilin Province, **C** Xiamen City, and **D** Chuxiong Prefecture

The number of reported TB cases in Wuhan City and Jilin Province showed a decreasing annual trend, while the number of reported TB cases in Xiamen City and Chuxiong Prefecture showed a slight fluctuation trend (Fig. 5). The incidence in the student population was distinctly low during the winter holidays (January–February, approximately 30 days) and summer vacation (July–August, approximately 60 days), with one or two distinct peaks after returning to school (the remaining months of the year). There were slight differences between regions in the time of occurrence of these peaks: Wuhan (March and September–October), Xiamen (March and October), Jilin (April and September), and Chuxiong (April and October). However, for the non-student population, there were no clear lows or peaks.

**Fig. 6:**
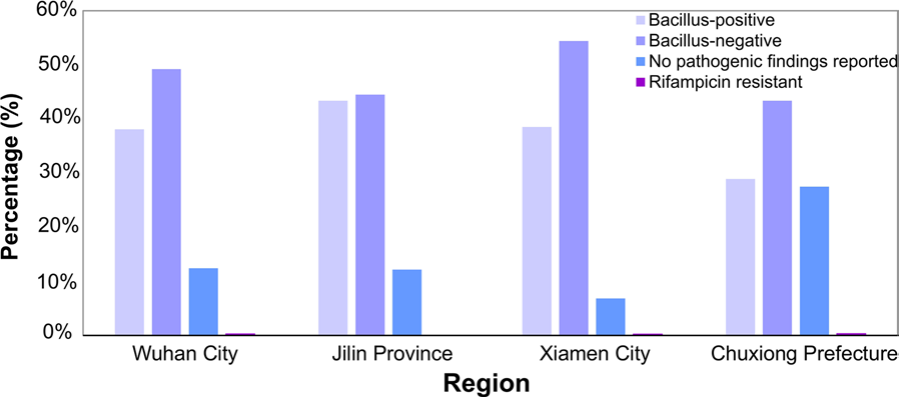
Proportions of patients reporting pulmonary tuberculosis in the four study areas

Most cases had either positive or negative pathogen results (87.3%), and the ratio was 1∶1.13. The proportion of cases without pathogenic findings was 12.6%; rifampicin resistant results accounted for 0.1%. The number of pathogen positive and negative cases was essentially the same in Jilin Province, while the other three regions reported more pathogen negative cases than positive. The proportion of patients without pathogenic findings was the lowest in Xiamen City and the highest in Chuxiong Prefecture. Very few cases of resistance to rifampicin were reported in any region (Fig. 6).

### Curve fitting

**Fig. 7:**
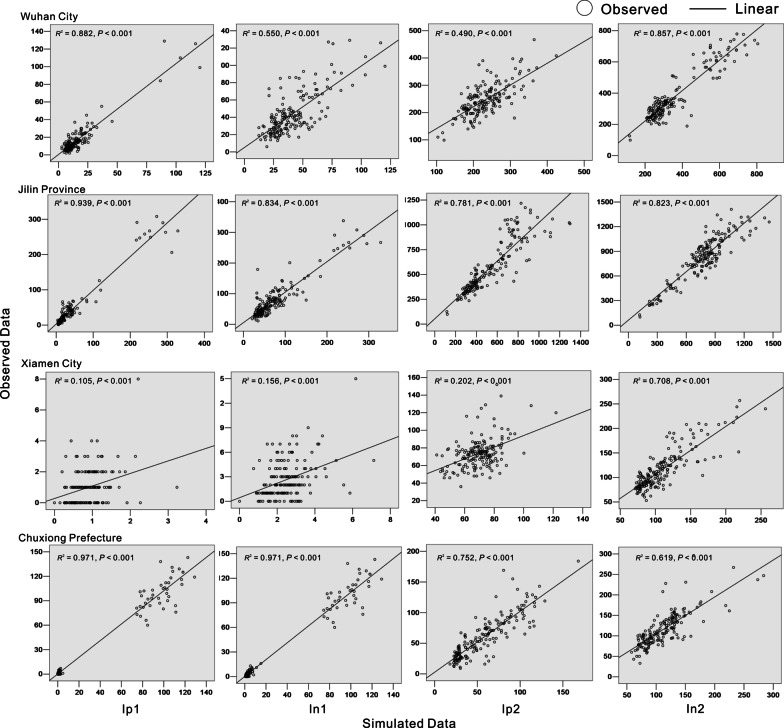
Plot of goodness-of-fit results for the non-seasonal model (Model A)

**Fig. 8:**
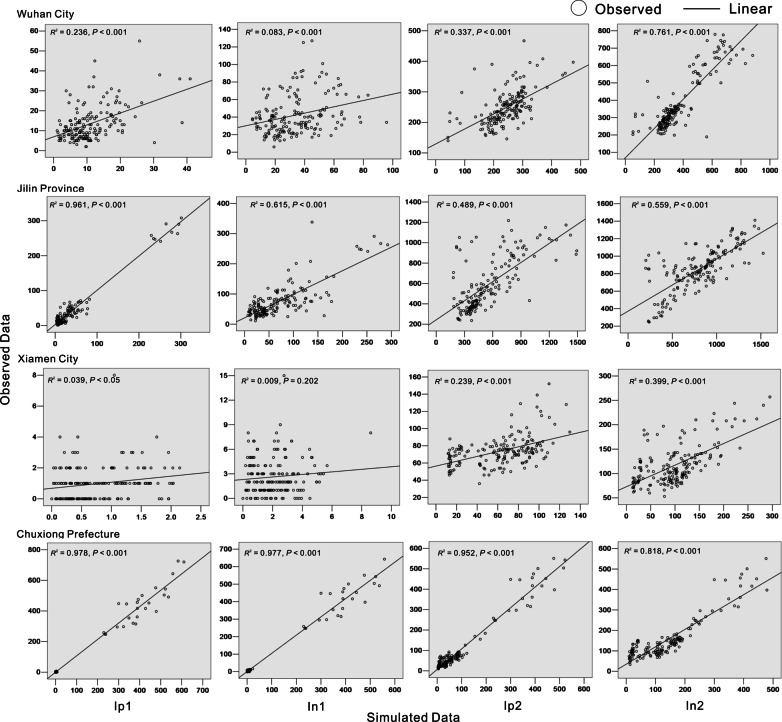
Plot of goodness-of-fit results for the seasonal model (Model B)

We conducted goodness-of-fit tests for the two models based on the case datasets from the four regions (Wuhan City, Jilin Province, Chuxiong Prefecture, and Xiamen City) (Figs. 7 and 8). *R*^2^ values were calculated for the four model groups (pathogen positive cases in the student group, *I*_*p*1_; pathogen negative cases in the student group, *I*_*n*1_; pathogen positive cases in the non-student group, *I*_*p*2_; and pathogen negative cases in the non-student group, *I*_*n*2_). The values showed that, although the two established TB models fitted well with the trend of TB incidence rates (Table 2), Model A had better fitting results than Model B.

**Table 2:** Goodness-of-fit test results of the two models (Models A and B) in the study areas

Region	*I*_*p*1_		*I*_*n*1_		*I*_*p*2_		*I*_*n*2_	
	*R*^2^	*P*	*R*^2^	*P*	*R*^2^	*P*	*R*^2^	*P*
Model A								
Wuhan City	0.882	< 0.001	0.55	< 0.001	0.49	< 0.001	0.857	< 0.001
Jilin Province	0.939	< 0.001	0.834	< 0.001	0.781	< 0.001	0.823	< 0.001
Xiamen City	0.105	< 0.001	0.156	< 0.001	0.202	< 0.001	0.708	< 0.001
Chuxiong Prefecture	0.971	< 0.001	0.971	< 0.001	0.752	< 0.001	0.619	< 0.001
Model B								
Wuhan City	0.236	< 0.001	0.083	< 0.001	0.337	< 0.001	0.761	< 0.001
Jilin Province	0.961	< 0.001	0.615	< 0.001	0.489	< 0.001	0.559	< 0.001
Xiamen City	0.039	0.009	0.009	0.202	0.239	< 0.001	0.399	< 0.001
Chuxiong Prefecture	0.978	< 0.001	0.977	< 0.001	0.952	< 0.001	0.818	< 0.001

Correlation between the simulated and observed data was tested using *R*_2_ and *p* values. We divided all the compartments representing active diseases (*I*) into four occupational compartments: pathogen positive students (*I*_*p*1_ subscript), pathogen positive non-students (*I*_*p*2_ subscript), pathogen negative students (*I*_*n*1_ subscript) and pathogen negative non-students (*I*_*n*2_ subscript)

### Transmissibility for interactions among the four groups

**Fig. 9:**
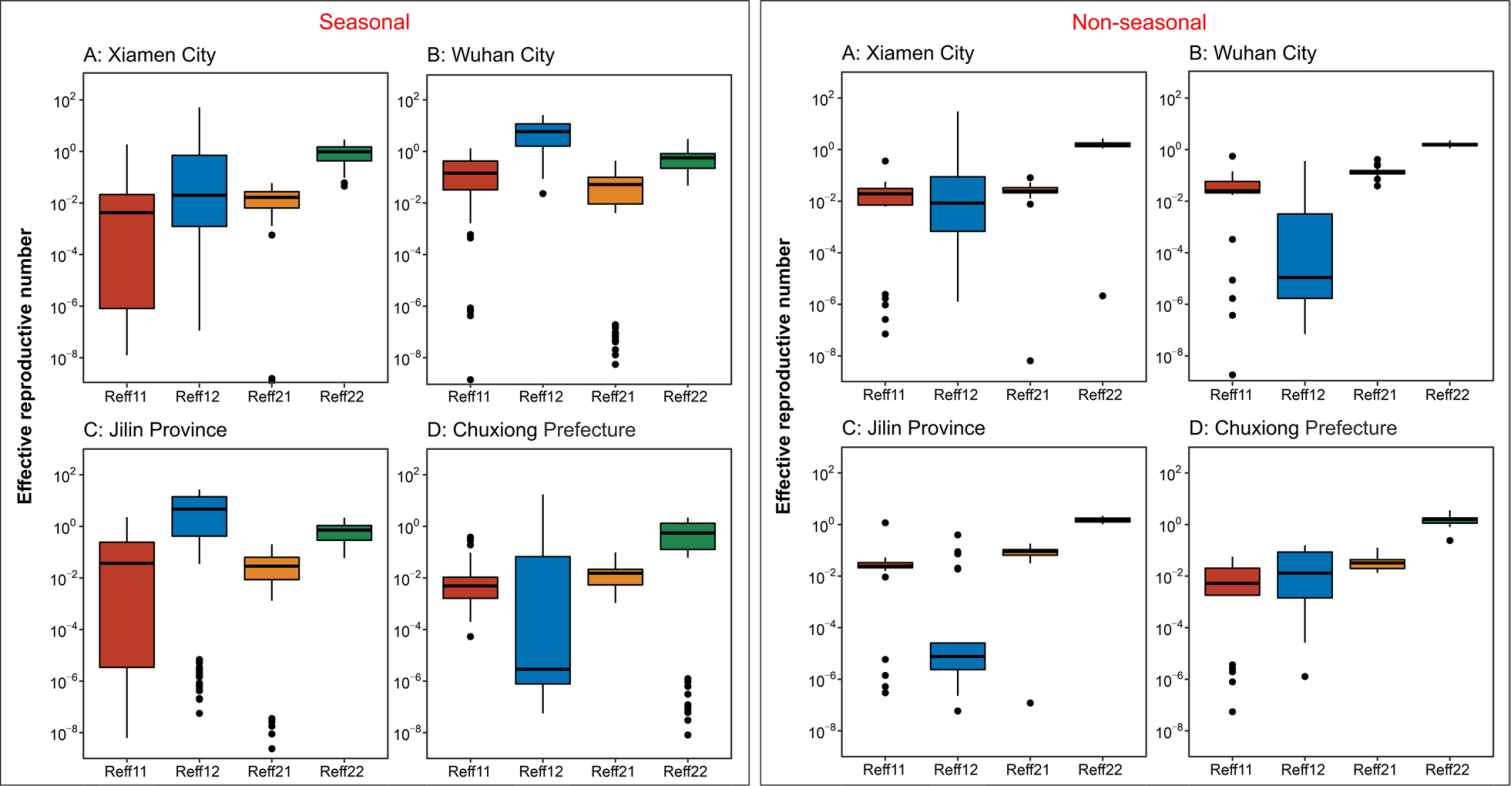
The chart of effective regeneration number plotted according to the two models

The results of *R*_*eff*_ among and between the different populations in each region are shown in Fig. 9.

In Wuhan City, the median *R*_*eff*_ for TB among the mixed population was 1.79 (IQR: 1.56–2.02). Most TB transmissions occurred due to the high transmission in non-student populations, including among non-student populations [median *R*_*eff*22_ was 1.57 (IQR: 1.41–1.72)] and non-student-to-student populations [median *R*_*eff*21_ was 0.14 (IQR: 0.11–0.15)], with a median *R*_*eff*2_ of 1.71(IQR: 1.54–1.87). The values of *R*_*eff*22_ and *R*_*eff*21_ slowly descended from 2005 to 2018. The values of *R*_*eff*12_ and *R*_*eff*11_ were nearly zero excluding in 2006 (*R*_*eff*12_ was 6.39, *R*_*eff*11_ was 0.19) and 2013 (*R*_*eff*11_ was 0.58) (Table 3).

**Table 3:** *R*_*eff*_ of Model A between students and non-students (Wuhan City)

Year	*R*_*eff*11_	*R*_*eff* 12_	*R*_*eff* 22_	*R*_*eff* 21_	*R*_*eff1*_	*R*_*eff2*_
2005 (asc)	0.04	0.00	2.12	0.24	0.04	2.37
2005 (des)	0.08	0.00	1.73	0.13	0.08	1.87
2006 (asc)	0.05	0.36	1.62	0.16	0.41	1.79
2006 (des)	0.14	0.00	1.75	0.16	0.14	1.91
2007 (asc)	0.06	0.00	1.86	0.14	0.07	2.00
2007(des)	0.04	0.00	1.49	0.13	0.04	1.62
2008 (asc)	0.02	0.00	2.27	0.42	0.02	2.68
2008 (des)	0.02	0.00	1.41	0.14	0.02	1.55
2009 (asc)	0.10	0.00	1.79	0.27	0.10	2.05
2009 (des)	0.00	0.00	1.12	0.14	0.00	1.26
2010 (asc)	0.06	0.00	1.75	0.20	0.06	1.94
2010 (des)	0.03	0.00	1.20	0.12	0.03	1.32
2011 (asc)	0.03	0.00	1.62	0.15	0.03	1.77
2011(des)	0.02	0.00	1.43	0.11	0.02	1.54
2012 (asc)	0.02	0.00	1.57	0.10	0.03	1.67
2012 (des)	0.02	0.00	1.45	0.08	0.02	1.53
2013 (asc)	0.55	0.01	1.61	0.08	0.56	1.69
2013 (des)	0.03	0.00	1.57	0.12	0.03	1.69
2014 (asc)	0.02	0.00	1.59	0.12	0.02	1.71
2014 (des)	0.02	0.00	1.25	0.12	0.02	1.37
2015 (asc)	0.00	0.02	1.57	0.15	0.02	1.72
2015 (des)	0.02	0.00	1.29	0.09	0.02	1.38
2016 (asc)	0.00	0.00	1.62	0.13	0.00	1.75
2016 (des)	0.06	0.00	1.41	0.15	0.06	1.56
2017 (asc)	0.11	0.01	1.71	0.16	0.11	1.87
2017 (des)	0.00	0.03	1.36	0.12	0.03	1.47
2018 (asc)	0.00	0.07	1.56	0.04	0.07	1.60
2018 (des)	0.02	0.00	1.21	0.07	0.02	1.28
Median	0.06	0.02	1.57	0.14	0.07	1.71
P25	0.02	0.00	1.41	0.11	0.02	1.54
P75	0.06	0.00	1.72	0.15	0.07	1.87

*R*_*eff*11_ denotes the transmissibility of MTB from student cases to student cases. *R*_*eff* 12_ denotes the transmissibility of MTB from student cases to non-student cases. *R*_*eff* 21_ denotes the transmissibility of MTB from non-student cases to student cases. *R*_*eff* 22_ denotes the transmissibility of MTB from the non-student cases to non-student cases. *R*_*eff*1_ represents the transmissibility of the population of students with active TB cases (sum of *R*_*eff*11_ and *R*_*eff* 12_), whereas *R*_*eff* 2_ represents the transmissibility of the population of non-student active TB cases (sum of *R*_*eff* 22_ and *R*_*eff* 21_)

asc denotes the ascending *R*_*eff*_ (*R*_*eff*(*asc*)_). des denotes the descending *R*_*eff*_ (*R*_*eff*(*des*)_)

In Jilin Province, the median *R*_*eff*_ for TB among the mixed population was 1.75 (IQR: 1.37–2.02). Most TB transmission occurred due to the high transmission in the non-student population, including among non-student populations [median *R*_*eff*22_ was 1.57, (IQR: 1.27–1.77)] and from non-student-to-student populations [median *R*_*eff*21_ was 0.09, (IQR: 0.07–0.11)], with a median *R*_*eff*2_ of 1.66 (IQR: 1.35–1.89). The *R*_*eff*21_ and *R*_*eff*12_ values maintained stable fluctuations at values lower than 1 from 2007 to 2019. The value of *R*_*eff*11_ was nearly zero excluding in 2009, when it reached 1.19 (*R*_*eff*(*asc*)_ 1.17, *R*_*eff*(*des*)_ 0.02) (Table 4).

**Table 4:** *R*_*eff*_ of Model A between students and non-students (Jilin Province)

Year	*R*_*eff*11_	*R*_*eff* 12_	*R*_*eff* 22_	*R*_*eff* 21_	*R*_*eff1*_	*R*_*eff2*_
2007 (asc)	0.00	0.00	1.27	0.03	0.00	1.30
2007 (des)	0.01	0.00	1.22	0.11	0.01	1.33
2008 (asc)	0.05	0.00	1.80	0.18	0.05	1.98
2008 (des)	0.00	0.00	1.23	0.11	0.00	1.35
2009 (asc)	1.17	0.02	1.60	0.00	1.19	1.60
2009 (des)	0.02	0.09	1.16	0.09	0.10	1.26
2010 (asc)	0.02	0.00	1.53	0.12	0.02	1.65
2010 (des)	0.00	0.02	1.25	0.09	0.02	1.34
2011 (asc)	0.05	0.00	1.99	0.15	0.05	2.14
2011 (des)	0.02	0.00	1.28	0.08	0.02	1.36
2012 (asc)	0.03	0.00	1.68	0.10	0.03	1.78
2012 (des)	0.02	0.00	1.25	0.06	0.02	1.31
2013 (asc)	0.03	0.00	1.84	0.09	0.03	1.93
2013 (des)	0.03	0.00	1.68	0.09	0.03	1.76
2014 (asc)	0.04	0.00	2.02	0.11	0.04	2.12
2014 (des)	0.02	0.07	1.54	0.05	0.10	1.59
2015 (asc)	0.04	0.00	2.04	0.10	0.04	2.13
2015 (des)	0.02	0.00	1.56	0.06	0.02	1.62
2016 (asc)	0.04	0.00	2.16	0.13	0.04	2.30
2016 (des)	0.03	0.00	1.58	0.06	0.03	1.64
2017 (asc)	0.03	0.00	2.02	0.09	0.03	2.11
2017 (des)	0.03	0.00	1.54	0.09	0.03	1.63
2018 (asc)	0.02	0.08	1.60	0.06	0.10	1.66
2018 (des)	0.02	0.00	1.48	0.11	0.02	1.59
2019 (asc)	0.00	0.40	1.40	0.09	0.40	1.49
2019 (des)	0.02	0.00	1.04	0.09	0.02	1.13
Median	0.07	0.03	1.57	0.09	0.09	1.66
P25	0.02	0.00	1.27	0.07	0.02	1.35
P75	0.03	0.00	1.77	0.11	0.05	1.89

*R*_*eff*11_ denotes the transmissibility of MTB from student cases to student cases. *R*_*eff* 12_ denotes the transmissibility of MTB from student cases to non-student cases. *R*_*eff* 21_ denotes the transmissibility of MTB from non-student cases to student cases. *R*_*eff* 22_ denotes the transmissibility of MTB from the non-student cases to non-student cases. *R*_*eff*1_ represents the transmissibility of the population of students with active TB cases (sum of *R*_*eff*11_ and *R*_*eff* 12_), whereas *R*_*eff* 2_ represents the transmissibility of the population of non-student active TB cases (sum of *R*_*eff* 22_ and *R*_*eff* 21_)

asc denotes the ascending *R*_*eff*_ (*R*_*eff*(*asc*)_). des denotes the descending *R*_*eff*_ (*R*_*eff*(*des*)_)

In Chuxiong Prefecture, the median *R*_*eff*_ of TB among the mixed population was 1.68 (IQR: 1.20–1.96). Most TB transmissions occurred due to the high transmission in non-student populations with a median *R*_*eff*22_ 1.59 (IQR: 1.14–1.80), and the other three values (*R*_*eff*11_, *R*_*eff*21_, *R*_*eff*12_) were nearly zero each year. The values of *R*_*eff*2_ and *R*_*eff*1_ fluctuated smoothly from 2008 to 2018, with a median *R*_*eff*2_ of 1.63 (IQR: 1.17–1.82) and a median *R*_*eff*1_ of 0.05 (IQR: 0.02–0.09), respectively (Table 5).

**Table 5:** *R*_*eff*_ of Model A between students and non-students (Chuxiong Prefecture)

Year	*R*_*eff*11_	*R*_*eff* 12_	*R*_*eff* 22_	*R*_*eff* 21_	*R*_*eff1*_	*R*_*eff2*_
2008 (des)	0.01	0.00	0.80	0.02	0.01	0.82
2009 (asc)	0.02	0.01	1.96	0.02	0.03	1.98
2009 (des)	0.00	0.04	0.87	0.02	0.05	0.89
2010 (asc)	0.02	0.00	1.97	0.03	0.02	2.01
2010 (des)	0.00	0.00	1.04	0.02	0.01	1.06
2011 (asc)	0.01	0.00	1.52	0.04	0.01	1.56
2011 (des)	0.00	0.08	1.11	0.01	0.08	1.12
2012 (asc)	0.02	0.00	1.26	0.03	0.02	1.29
2012 (des)	0.01	0.05	1.72	0.02	0.06	1.74
2013 (asc)	0.00	0.01	0.24	0.05	0.02	0.29
2013 (des)	0.00	0.09	1.71	0.02	0.09	1.73
2014 (asc)	0.02	0.00	1.14	0.04	0.02	1.17
2014 (des)	0.00	0.09	1.78	0.04	0.09	1.82
2015 (asc)	0.03	0.02	2.82	0.13	0.05	2.95
2015 (des)	0.03	0.00	1.73	0.05	0.03	1.78
2016 (asc)	0.06	0.00	3.58	0.09	0.06	3.66
2016 (des)	0.00	0.13	1.52	0.02	0.13	1.54
2017 (asc)	0.00	0.16	1.80	0.00	0.16	1.80
2017 (des)	0.00	0.09	1.58	0.03	0.09	1.61
2018 (asc)	0.03	0.00	2.01	0.12	0.04	2.12
2018 (des)	0.00	0.09	1.26	0.04	0.09	1.30
Median	0.01	0.04	1.59	0.04	0.05	1.63
P25	0.00	0.00	1.14	0.02	0.02	1.17
P75	0.02	0.09	1.80	0.04	0.09	1.82

*R*_*eff*11_ denotes the transmissibility of MTB from student cases to student cases. *R*_*eff* 12_ denotes the transmissibility of MTB from student cases to non-student cases. *R*_*eff* 21_ denotes the transmissibility of MTB from non-student cases to student cases. *R*_*eff* 22_ denotes the transmissibility of MTB from the non-student cases to non-student cases. *R*_*eff*1_ represents the transmissibility of the population of students with active TB cases (sum of *R*_*eff*11_ and *R*_*eff* 12_), whereas *R*_*eff* 2_ represents the transmissibility of the population of non-student active TB cases (sum of *R*_*eff* 22_ and *R*_*eff* 21_)

asc denotes the ascending *R*_*eff*_ (*R*_*eff*(*asc*)_). des denotes the descending *R*_*eff*_ (*R*_*eff*(*des*)_)

In Xiamen City, we excluded data analysis in 2019 for only 3 months data collection from January to March, which was not a valid TB representation for the entire year. Except that the median *R*_*eff*_ for TB among the mixed population was 1.67 (IQR: 1.40–1.93). Most TB transmissions of occurred due to the high transmission in non-student populations, with a median *R*_*eff*22_ of 1.58 (IQR: 1.32–1.80). *R*_*eff*2_ values slowly decreased between 2005 and 2018, with a median *R*_*eff*2_ of 1.61 (IQR: 1.35–1.85). Although there were several values of *R*_*eff*12_ higher than 0.10 in student-non-student transmission (*R*_*eff* (*des*)_ in 2005, 2012, 2016, and *R*_*eff* (*asc*)_ in 2006, 2008, 2010), the overall transmissibility was annual decreasing with a median *R*_*eff*12_ of 0.04 (IQR: 0.00–0.07) (Table 6).

**Table 6:** *R*_*eff*_ of Model A between students and non-students (Xiamen City)

Year	*R*_*eff*11_	*R*_*eff* 12_	*R*_*eff* 22_	*R*_*eff* 21_	*R*_*eff1*_	*R*_*eff2*_
2005 (asc)	0.04	0.00	2.21	0.03	0.04	2.24
2005 (des)	0.00	0.22	2.03	0.03	0.22	2.06
2006 (asc)	0.02	0.11	2.71	0.01	0.13	2.72
2006 (des)	0.02	0.00	1.77	0.05	0.02	1.83
2007 (asc)	0.04	0.00	1.81	0.04	0.04	1.84
2007 (des)	0.03	0.01	2.00	0.04	0.04	2.04
2008 (asc)	0.00	0.15	1.86	0.03	0.15	1.89
2008 (des)	0.03	0.00	1.54	0.03	0.03	1.57
2009 (asc)	0.00	0.09	1.75	0.02	0.09	1.77
2009 (des)	0.04	0.01	1.69	0.05	0.05	1.73
2010 (asc)	0.04	0.15	1.60	0.03	0.19	1.62
2010 (des)	0.03	0.01	1.33	0.02	0.04	1.36
2011 (asc)	0.03	0.00	2.01	0.05	0.03	2.06
2011 (des)	0.01	0.00	1.39	0.03	0.01	1.42
2012 (asc)	0.02	0.00	1.36	0.02	0.02	1.39
2012 (des)	0.00	0.12	1.31	0.03	0.12	1.34
2013 (asc)	0.06	0.00	1.79	0.08	0.06	1.87
2013 (des)	0.02	0.00	1.28	0.02	0.02	1.29
2014 (asc)	0.01	0.04	1.16	0.01	0.05	1.18
2014 (des)	0.01	0.02	1.19	0.03	0.03	1.22
2015 (asc)	0.02	0.00	1.48	0.02	0.02	1.50
2015 (des)	0.01	0.02	1.21	0.05	0.03	1.27
2016 (asc)	0.02	0.01	1.34	0.02	0.03	1.37
2016 (des)	0.00	0.10	1.11	0.01	0.10	1.12
2017 (asc)	0.02	0.00	1.38	0.02	0.02	1.40
2017 (des)	0.01	0.06	1.43	0.01	0.07	1.45
2018 (asc)	0.02	0.01	1.32	0.02	0.03	1.35
2018 (des)	0.02	0.00	1.22	0.02	0.02	1.25
Median	0.02	0.04	1.58	0.03	0.06	1.61
P25	0.01	0.00	1.32	0.02	0.03	1.35
P75	0.03	0.07	1.80	0.03	0.07	1.85

*R*_*eff*11_ denotes the transmissibility of MTB from student cases to student cases. *R*_*eff* 12_ denotes the transmissibility of MTB from student cases to non-student cases. *R*_*eff* 21_ denotes the transmissibility of MTB from non-student cases to student cases. *R*_*eff* 22_ denotes the transmissibility of MTB from the non-student cases to non-student cases. *R*_*eff*1_ represents the transmissibility of the population of students with active TB cases (sum of *R*_*eff*11_ and *R*_*eff* 12_), whereas *R*_*eff* 2_ represents the transmissibility of the population of non-student active TB cases (sum of *R*_*eff* 22_ and *R*_*eff* 21_)

asc denotes the ascending *R*_*eff*_ (*R*_*eff*(*asc*)_). des denotes the descending *R*_*eff*_ (*R*_*eff*(*des*)_)

A similar transmission relationship among and between student and non-student populations was calculated in Model B. However, the model revealed exceedingly high values of *R*_*eff*_ over many years in the four regions, which indicates that the findings of Model B may be unsuitable to show the characteristics of TB. Additional details of the results are provided in Additional file 3: Tables S3, Additional file 4: Table S4, Additional file 5: Table S5, Additional file 6: Table S6.

### Cumulative incidence rate after the knock-out-pathways, ***β***_11_, ***β***_12_, ***β***_22_, and ***β***_21_

**Fig. 10:**
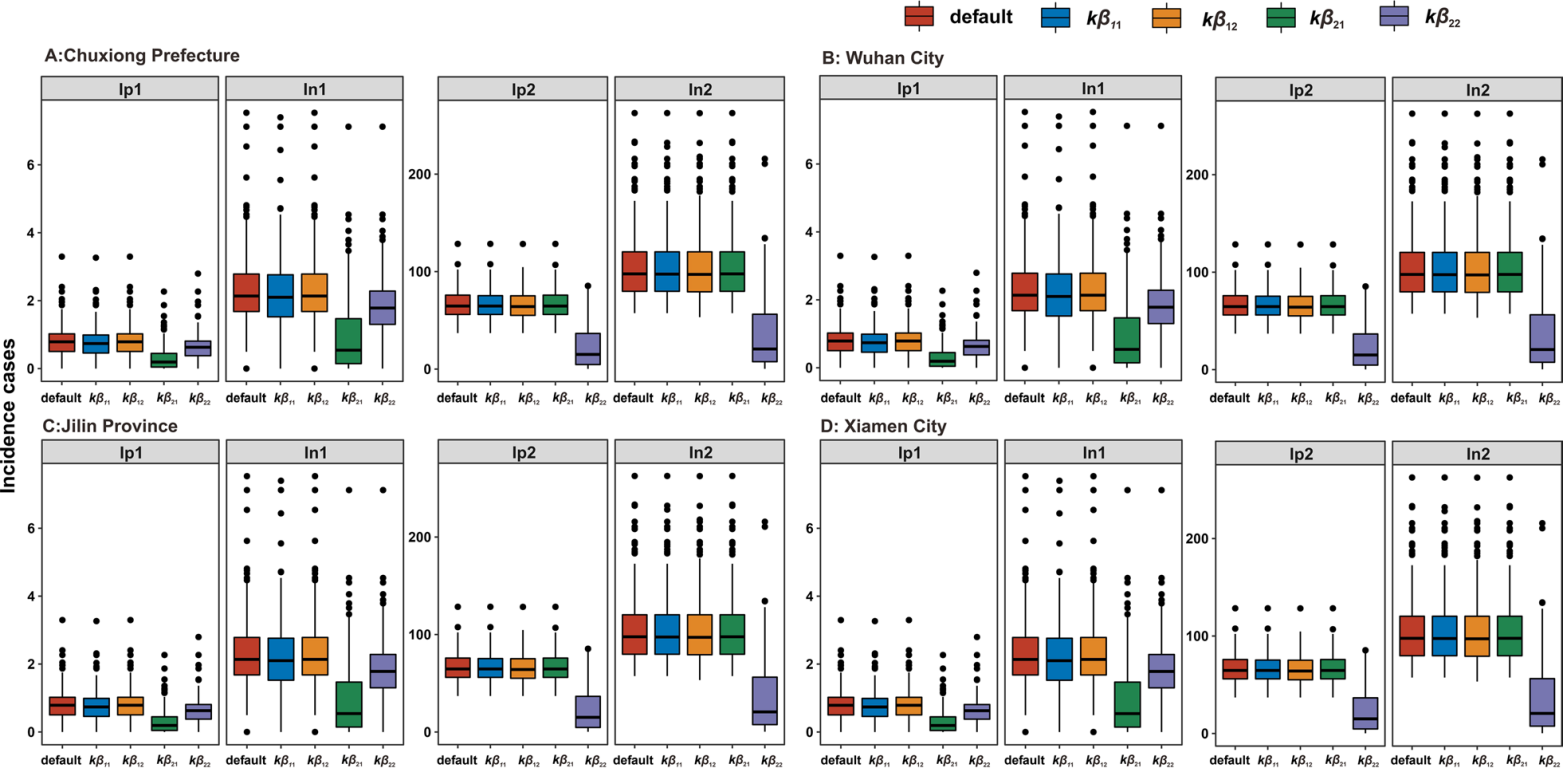
Knockout analysis in the four study areas: **A** Wuhan City, **B** Jilin Province, **C** Xiamen City, and **D** Chuxiong Prefecture. The subscripts refer to the occupation of the students (_1_ subscript) or non-students (_2_ subscript). The initial state is denoted as the default. The performance of the knockout for each of the transmission pathway was determined by setting the beta value to zero. For example, k_b11_ stands for getting rid of the student-to-students’ transmission pathway

According to the knock-out results (Fig. 10), the number of TB cases among students was significantly reduced by more than half (60–70%) when the transmissibility of non-student-to-student populations (*β*_21)_ was knocked out. When TB transmission among non-students (*β*_22)_ was blocked, the number of TB cases was reduced by approximately 67% (65–70%) among non-students and by approximately 28% (25–30%) among students. There was only a 5% reduction (2–12%) among students when TB transmission among students (*β*_11)_ was blocked, and TB reported cases had barely changed (less than 1%) while TB transmission from non-student-to-students (*β*_12)_ was blocked.

## Discussion

This study is the first to address the occupational-specific transmission dynamics of TB and emphasise the importance of control between student groups, which can increase our understanding of the characteristics of TB transmission in different occupational groups.

### Analysis of epidemiological characteristics

The incidence rate of TB decreased in the study regions, which is in good agreement with previous global reports [3], but was unevenly distributed between these four regions. This phenomenon may be attributed to several reasons. First, the inclusion of previously untreated patients in the management after several years of continuous active screening led to a certain decline in the number of subsequent patient detection cases. Second, since 2017, a nationwide survey of underreporting and under-registration of TB [58] and a diagnostic review [59] were carried out, which improved the quality of TB reporting and diagnosis in each region. However, the reported incidence rate of TB is still higher in areas less economically developed than in the west, such as Chuxiong Prefecture, although the attention and support of governments and health administrations, as well as the support for precise health poverty alleviation have been undertaken at all levels [9].

Remarkably, the reported incidence of TB in the student population has increased. The reported data also confirmed that the proportion of student patients has increased from 4.0% in 2015 to 6.2% in 2019, with a difference of 2.2% [9]. This is mainly due to the fact that early warning of individual cases of TB in schools has been included in the National Automatic Early Warning System for Infectious Diseases since July 2018 [60]. Furthermore, disease control agencies at all levels have strengthened the information verification process of school-age patients and improved the sensitivity of the surveillance of student patients [61]. Schools have also strengthened medical examinations and handled clusters of TB outbreaks [62]. Our results highlight an obvious incidence peak among students at the beginning of the semester. There are several explanations for this observation. Students are in close contact with social residents and are exposed to TB patients in the community during holidays. Considering LTBI [8, 63], students infected with MTB on vacation do not become ill immediately, but become ill after returning to school when they are exposed to several inducements, including high pressure and cold, among others. Farmers always had the highest reported incidence rates. However, this is not surprising if we consider that the rural population represents most of the total population of China, and the allocation of medical and health resources in rural areas is inadequate, resulting in unequal access to medical resources for urban and rural residents [13]. Furthermore, it may be related to the lower level of education of farmers, poorer living conditions, and lack of awareness of health protection [64].

The diagnosis results in the study areas, which had a low pathogen positive rate of less than 50%. A previous report showed the pathogen positive rate for PTB reported in China in 2020 was 57%, up from 45.03% in 2019 [3]. However, a gap still exists when this is compared with surveillance results based on laboratory pathogenic diagnostic evidence in other countries worldwide. Both the TB laboratory diagnostic and the TB imaging detection capacity need to be improved in primary care institutions in China, which is consistent with the outcomes of one diagnostic and therapeutic survey on TB sentinel medical institutions [65] and on the current status of TB diagnostic capacity at county-level TB sentinel medical institutions in China [66]. To achieve the goal of "reaching a pathogenic positivity rate of more than 50% by 2022" as required by the Action Plan to Stop TB (2019–2022) [67], it is still necessary to continue to strengthen the quality of laboratory work [68].

### Analysis of TB transmission dynamics characteristics 

In this study, two mathematical models of TB were constructed according to the transmission characteristics of TB: Models A and B. Although there may be seasonal fluctuations in the actual incidence of TB in some areas, Model A fitted better than Model B. Therefore, we believe that the analysis results of Model A can better reflect the real situation of TB incidence.

Therefore, the following interpretations were made according to the results of the *R*_*eff*_ calculation of Model A and results of a knock-out analysis:

A) Overall, the average values of *R*_*eff*_ in the four regions showed that a single TB case could effectively spread to one or two people. TB transmissibility among non-students (*R*_*eff*2_) was 23.30 times (IQR: 1.94–7.24) higher than among students (*R*_*eff*1_). TB transmission remained dominant in the non-student population. This finding also existed in the knock-out analysis. Transmission among non-students increased the number of reported TB cases in all four groups (67% in non-students and 28% in students). The non-student population was large, and included 17 occupations, different locations with active cases, and a wide range of age groups. In high-burden areas, such as China, most TB transmission occurs outside the home (< 20% of household transmission) which is not necessarily attributable to known close contacts [69, 70]. The probability of TB transmission to others by a TB patient is determined by many factors, including socioeconomic, environmental, high or low regional disease burden, infectiousness of the case, MTB strain, and host susceptibility. Determining the specific site of TB transmission outside the home is difficult. The potential for airborne transmission even during brief contact, combined with variable incubation periods, makes it exceptionally difficult to establish a specific TB transmission link. Despite these challenges, certain specific settings have been identified as important contributors to TB risk, such as nasal transmission [71–74], hospital-associated transmission [75], homeless shelters [76], prisons [77, 78], public transportation [79], churches [80], schools [69, 81] and slums [82–84]. This is precisely because the places where students study and live are close, providing good conditions for the spread of TB; therefore, the implementation of TB control policies in schools is especially important. The presence of these factors has contributed to the high rates of acquired TB in this group over the years.

Furthermore, the concentration of TB transmission in certain settings and subpopulations also leads to heterogeneity of transmission, which can serve to increase *R*_*eff*_ and may make it more difficult to control transmission [85]. Moreover, adults in their most active age groups are more likely to be infected with TB due to their close contact with each other [86]. To explain why transmission among the non-student populations increased the number of infected patients among non-students, it may be assumed that household and unnoticed transmissions in the community contribute simultaneously [87].

B) The results from knock-out analysis indicated that non-student-to-student transmission increased the number of reported TB cases in the student group (either pathogen positive or negative), and transmission among non-students increased the number of reported TB cases in all the four groups. There may be several reasons for this. First, the home–school transmission route may be one of the reasons. TB is actively transmitted by household exposure [88], and a prospective case–control study found that previous exposure to TB in a household could cause an infected student to spread TB to their classmates [89]. Second, we believe that the school community transmission route is important due to increased exposure to other occupations during vacations.

C) Although TB transmission is spread mainly by non-students, the transmissibility of student-to-non-students in some years and in some regions, is particularly high, such as the *R*_*eff*12_ of Chuxiong Prefecture in 2016 (*R*_*eff*12_: 0.13), 2017 (*R*_*eff*12_: 0.16), and that of Wuhan City in 2006 (*R*_*eff*12_: 0.36), etc. This could be due to TB outbreaks in schools [90]. Once TB transmission occurs in schools, the spread of TB will exceed beyond the public due to the frequent contact between students and cause widespread TB in schools. Due to this particularity of TB school transmission, the TB reporting system of China is more sensitive to the population of student occupation. A national single-case warning system is used to identify the student tuberculosis patients. When a student is diagnosed, close contacts screening, isolation and treatment of the TB patients are implemented in the shortest time. These measures make the control of student TB outbreaks much more effective, and then reduce the tuberculosis cases of this outbreak. But in the real world, if the epidemic was not dealt with promptly, a widespread TB outbreak in schools will be inevitable.

### Prevention and control of TB among students

The relevant authorities must continue to strengthen the prevention and control of TB in student populations in the future [91]. There are shortcomings at all levels, including schools, medical institutions and TB control institutions, and improvements are needed. For schools, the implementation of a system to trace the causes of absence from school to detect patients in a timely and proactive manner is effective. Medical institutions should keep the epidemic information channels open with schools and TB control institutions, and provide timely information about confirmed students to schools and TB control institutions. TB prevention and control institutions should perform timely information verification and close contact follow-up after the detection of the infected student.

In addition, we suggest that more attention should be paid to men, farmers, and young and middle-aged people; and the bacteriological diagnosis of TB should be strengthened. More data collection from social contact surveys is required to provide information on how individual behaviors drive disease dynamics at the population level.

In particularly, several limitations may have influenced the results obtained. The first is selection bias due to inconsistency at the administrative levels in our study areas, which includes three cities and one province. The second is that we only included cases that were diagnosed as “bacteriologically confirmed positive or negative” and excluded those that were diagnosed as “rifampicin resistant” when processing the initial data. The latter could also contribute to TB transmission. Furthermore, complete immunity does not occur in patients with TB after recovery. However, partial immunity has been observed in previously infected individuals, which can prevent reinfection (risk ratio = 0.5) [92]. The last limitation of our methodology is that it was not possible to subdivide the 17 non-student occupations to better articulate the mechanisms of transmission between different occupations and quantify the impact of different non-student occupations on the student population.

## Conclusions

This study has the potential to improve our understanding of the features of TB transmission in different occupational groups. The transmission of MTB was high in non-student populations, and that in the non-student population was 23.30 times higher than in the student population. It had the strongest influence among non-student groups. It not only increases the incidence of TB among non-students, but also among students. The incidence of TB among students has been on the rise and is the fourth highest in occupational distribution (especially in economically developed areas with a high number of students), despite the incidence of TB in China showing a downward trend annually. The TB outbreak among students can rapidly improve the transmissibility of TB in a short time, which will affect the prevalence of TB in other groups. TB screening should be performed rigorously at the beginning of the school semesters, when returning to school, to detect patients with LTBI. This implies the need for the implementation of more control measures such as strengthening the school TB management efforts and timely management of identified TB-infected students, after the academic year begins.

## Supplementary Information

**Additional file 1: Table S1.** The basic information of the four regions.

**Additional file 2: Table S2.** Two different classifications of tuberculosis in National Notifiable Disease Surveillance System (NNDSS).

**Additional file 3: Table S3.** Estimation of transmissibility between students and non-students (Wuhan City).

**Additional file 4: Table S4.** Estimation of transmissibility between students and non-students (Jilin Province).

**Additional file 5: Table S5.** Estimation of transmissibility between students and non-students (Chuxiong Perfecture).

**Additional file 6: Table S6.** Estimation of transmissibility between students and non-students (Xiamen City).

## Data Availability

The datasets used and/or analysed during the current study are available from the corresponding author on reasonable request.

## Abbreviations

SEIR: Susceptible-exposed-symptomatic-recovered
*R*_*eff*_: Effective reproduction number
MTB: *Mycobacterium tuberculosis*
TB: Tuberculosis
PTB: Pulmonary tuberculosis
MDR-TB: Multidrug resistant tuberculosis
HIV: Human immunodeficiency virus
AIDS: Acquired immune deficiency syndrome
LTBI: Latent TB infections
NNDSS: National Notifiable Disease Surveillance System
IGRA: Interferon-gamma release assays
*R*_0_: Basic reproduction number
DOTS: Directly observed treatment and short course chemotherapy
